# A Comparative Study of Molecularly Imprinted Polypyrrole Architectures for Electrochemical Quartz Microbalance-Based Method Development for Geraniol Adsorption

**DOI:** 10.3390/polym18070804

**Published:** 2026-03-26

**Authors:** Greta Kaspute, Deivis Plausinaitis, Vilma Ratautaite, Evelina Vaicekauskaite, Arunas Ramanavicius, Urte Prentice

**Affiliations:** 1 Department of Nanotechnology, State Research Institute Center for Physical Sciences and Technology (FTMC), Sauletekio Ave. 3, LT-10257 Vilnius, Lithuania; greta.kaspute@ftmc.lt (G.K.); vilma.ratautaite@ftmc.lt (V.R.); 2 Department of Physical Chemistry, Institute of Chemistry, Faculty of Chemistry and Geosciences, Vilnius University (VU), Naugarduko Str. 24, LT-03225 Vilnius, Lithuania; deivis.plausinaitis@chf.vu.lt (D.P.); evelina.vaicekauskaite@chgf.stud.vu.lt (E.V.); arunas.ramanavicius@chf.vu.lt (A.R.)

**Keywords:** geraniol, molecularly imprinted polymers (MIPs), sensors, analytical chemistry, electrochemistry, conducting polymers, electrochemical deposition

## Abstract

Molecularly imprinted polymers (MIPs) are widely employed for selective adsorption of target molecules in sensing and separation applications. The architecture of MIP films can influence adsorption behavior, interfacial stability, and reusability, yet systematic investigations of these effects are limited. This study aimed to evaluate how different polypyrrole (PPy) MIP film architectures affect the adsorption, stability, and regeneration characteristics of geraniol-imprinted layers on gold electrodes. Geraniol-imprinted and non-imprinted PPy films were electropolymerized onto quartz crystal microbalance (QCM) substrates. Two film architectures were compared: (i) a single-layer geraniol-imprinted PPy film, and (ii) a double-layer film consisting of a non-imprinted PPy underlayer followed by a geraniol-imprinted layer. Film characterization was performed using cyclic voltammetry (CV) and electrochemical quartz crystal microbalance (EQCM) measurements. Adsorption–desorption cycles were conducted to assess mass uptake, signal stability, and regeneration performance. EQCM analysis revealed that the double-layer architecture exhibited enhanced frequency signal stability during repeated adsorption–desorption cycles compared to single-layer films, suggesting a stabilizing effect of the underlying non-imprinted PPy layer at the electrode interface. Geraniol-imprinted films demonstrated significantly higher mass uptake than non-imprinted controls, confirming the sensitivity provided by molecular imprinting. Single-layer films showed more variability in signal response and less consistent regeneration performance. The architecture of MIP films significantly affects adsorption behavior, stability, and regeneration on electrode surfaces. Incorporating a non-imprinted PPy underlayer can improve signal reproducibility and enhance the robustness of MIP-based sensing interfaces. These findings provide guidance for the rational design of MIP coatings for electrochemical sensors and QCM-active platforms.

## 1. Introduction

Today, phytotherapy has emerged as a valuable source for the discovery of novel therapeutic agents [1]. Herbal medicines are becoming important materials for drug discovery due to their broad therapeutic potential [2]. Among herbal products, essential oils (EOs), complex mixtures of volatile, lipophilic, and aromatic compounds, exhibit diverse biological activities, including antimicrobial, antioxidant, anti-inflammatory, and anticancer effects. This broad spectrum of potential biological effects makes them promising candidates for pharmaceutical development [3]. However, these materials face challenges, including poor solubility in water-based solutions (around 100 mg/L at 25 °C), low bioavailability, and instability under physiological conditions, which often limit their clinical efficacy [4].

To overcome the limitations of traditional herbal formulations and enhance therapeutic efficacy, advanced drug delivery systems are being actively explored [5]. Molecularly imprinted polymers (MIPs), synthetic receptors known for their high selectivity, stability, and molecular recognition capabilities, have shown great potential in various applications such as sensing technologies, extraction processes, catalysis, and drug delivery [6]. Beyond their analytical use, MIPs also serve as versatile model systems for studying molecular recognition and adsorption phenomena at electrochemical interfaces [7]. When integrated with electrochemical and gravimetric techniques, MIP films enable detailed investigation of adsorption behavior, film stability, and interfacial processes [8].

Despite these advantages, MIP design is challenging due to the molecular size, structure, and flexibility of target analytes, often resulting in heterogeneous binding sites and limited binding efficiency [9]. In addition, film thickness, mechanical stability, and interfacial adhesion can strongly influence adsorption reversibility and signal stability, particularly in electrochemical and QCM-based systems [10]. Recent innovations have shown potential to overcome these limitations, e.g., surface or epitope imprinting, and nanoscale MIPs with surface-accessible binding sites. While computational tools have shown promise in optimizing MIP design, their integration into the fabrication process remains limited [6,11,12,13,14].

Geraniol is a naturally occurring monoterpene alcohol widely used in pharmaceutical development as a functional excipient and, in some cases, as a bioactive compound. Owing to its antimicrobial, anti-inflammatory, and penetration-enhancing properties, it is primarily applied in topical and advanced drug-delivery formulations. It functions as a transdermal penetration enhancer [15] and exhibits potent antioxidant, anti-inflammatory, and anticancer properties [16], including pro-apoptotic effects against cancers of the prostate, liver, kidney, bowel, and skin [17,18,19]. Additionally, geraniol has demonstrated antibacterial activity against respiratory, dermatological, and foodborne pathogens [20,21], and shows potential for synergistic action in chemotherapeutic regimens [16]. Owing to its hydrophobicity and molecular flexibility, geraniol also represents a challenging target for molecular imprinting, making it suitable for investigating architecture-dependent adsorption behavior in MIP films [22]. Previous studies have reported QCM-based investigations of geraniol–MIP interactions, including a dual sensor array using MIP technology, which successfully distinguished citronellal and geraniol in citronella (*Cymbopogon nardus*) EO with high selectivity, as confirmed by principal component analysis (separability index: 38.996) [23]. Another study utilized an MIP synthesized from commercial silicone sealant and geraniol, applied via drop-casting onto a silver-electrode QCM, to achieve sensitive and selective detection at ppm levels in palmarosa (*Cymbopogon martinii*) EO, with results comparable to those obtained by gas chromatography [24]. While these studies demonstrate the feasibility of geraniol imprinting, systematic investigations into how MIP layer architecture influences adsorption stability and regeneration behavior remain limited.

In this context, the present work examines how MIP layer architecture influences adsorption behavior, interfacial stability, and regeneration characteristics at electrochemical interfaces. Two types of MIP architecture are compared in this study: single- and double-layer geraniol-imprinted polypyrrole films deposited on gold electrodes were investigated using electrochemical quartz crystal microbalance (EQCM) and cyclic voltammetry (CV). In the double-layer configuration, a non-imprinted polypyrrole underlayer is introduced beneath the imprinted film to serve as an interfacial stabilization layer at the electrode–polymer boundary. We hypothesize that the presence of this non-imprinted underlayer modulates polymer adhesion and charge-transfer properties at the gold surface, thereby influencing the signal frequency stability and the reversibility of adsorption, without directly contributing to molecular recognition. Rather than pursuing sensitivity enhancement, the double-layer architecture is employed here as a mechanistic model to decouple substrate–polymer interfacial effects from imprinting-driven adsorption behavior. By systematically comparing single- and double-layer architectures of identical imprinting chemistry, this study aims to clarify the role of the base layer in governing adsorption stability and regeneration phenomena in MIP-modified electrochemical and QCM-active interfaces.

## 2. Materials and Methods

### 2.1. Materials and Instrumentation

**Chemicals:** Pyrrole (98%, Alfa Aesar, Karlsruhe, Germany) was distilled before use for the preparation of MIPs. Acetonitrile (Carl Roth GmbH & Co., Karlsruhe, Germany) and geraniol (Sigma-Aldrich, St. Louis, MO, USA) were used as received. Lithium perchlorate (LiClO_4_) (Thermo Fisher Scientific, Bremen, Germany) was used for buffer preparation. First-grade, freshly distilled water (resistivity: 0.055 μS/cm at 25 °C) was used in all aqueous solutions. Ammonium hydroxide solution 25% (pure p.a. CAS No: 1336-21-6, Thermo Fisher Scientific, Warszawa, Poland), hydrogen peroxide ≥ 30% (p.a. CAS: 7722-84-1, Merck Life Science, Bratislava, Slovakia).

**Electrochemical instrumentation:** All electrochemical measurements were carried out using a Metrohm AutoLAB potentiostat/galvanostat (µAutolabIII/FRA2 µ3AUT71079, controlled by NOVA 2.1.3 software, EcoChemie, B.V., Utrecht, The Netherlands), and a Gamry Echem Analyst potentiostat/galvanostat (version 5.30, Warminster, PA, USA) [25]. A binary HP 1100 HPLC pump (model G1312A, Hewlett-Packard, Böblingen, Germany) was used for liquid handling and flow administration. A six-channel, two-position Rheodyne valve (IDEX Health & Science, Rohnert Park, CA, USA) was used for the sample administration in the system. A 5 MHz QSX 301 *Biolin Scientific* (Biolin Scientific AB, Västra Frölunda (Gothenburg), Sweden) sensor with a surface area of S = 0.79 cm^2^ was used as the working electrode for an electrochemical QCM (E-QCM), on the 0.20 cm^2^ acoustically active QCM area. A custom-made 3D-printed flow cell (volume: 350 μL) was used for the measurements. The sensor was connected to an oscillator circuit and controlled by a Maxtek RQCM (INFICON Inc., New York, NY, USA) device. Measurements were performed in real time under flow conditions (1 mL/min) [26].

### 2.2. Preparation of Solutions for Sensor Development

A series of solutions was prepared for electrochemical analyses.

Solution I: 0.1 mol/L LiClO_4_ in acetonitrile and distilled water.

Solution II: 0.05 mol/L pyrrole in Solution I. This solution was used to polymerize the pyrrole layer in double-layer formation and to develop NIP.

Solution III: pyrrole (0.05 mol/L) and geraniol (0.005 mol/L) in Solution I. This solution was used in MIP synthesis.

NIP and MIP were compared to geraniol rebinding.

### 2.3. Pretreatment of the Working Electrode Before MIP or NIP Electrochemical Deposition

The working electrode was pretreated for electrochemical deposition of polypyrrole (Ppy) and geraniol according to established protocols [27]. Electrochemical cleaning was performed in a 0.1 mol/L LiClO_4_ solution containing a redox probe to enable electron-transfer cycling (such as dissolved oxygen species), by cycling the potential between −500 mV and +1000 mV vs. Ag/AgCl at a sweep rate of 100 mV/s, repeated up to a maximum of 10 cycles to avoid degradation of the gold-coated QCM surface. During electrochemical cleaning, adsorbed organic contaminants (commonly sulfur-containing species from air exposure on gold surfaces) undergo reduction at negative potentials and oxidation at positive potentials, removing them from the electrode surface and diffusing them into the bulk solution [28]. Cleaning was continued until a stable CV response was obtained, indicating a reproducible and contaminant-free Au surface for subsequent polymer deposition.

### 2.4. CV Parameters in MIP and NIP Preparation

Electrochemical experiments were performed in a three-electrode electrochemical cell with a 5 MHz gold-coated QCM sensor as the working electrode, a platinum counter electrode, and an Ag/AgCl pseudo-reference electrode. All parameters for the deposition of two types of MIP architecture: single- and double-layer geraniol-imprinted polypyrrole and non-imprinted polypyrrole (NIP) films are presented in Table 1.

The number of electropolymerization cycles was selected to ensure continuous, mechanically stable film formation for each configuration, rather than to achieve identical total film thickness, as the focus of this study is on architecture-dependent interfacial behavior rather than absolute adsorption capacity.

### 2.5. QCM Solutions and Parameters

For QCM measurements, two reference solutions were prepared. Solution A, a 50:50 (*v*/*v*) mixture of acetonitrile and water, was used to establish baseline frequency stability. Solution B was prepared by dissolving 0.05 mol/L geraniol in Solution A and was used to study analyte–polymer interactions. Comparing frequency shifts between these two conditions enabled quantitative evaluation of geraniol adsorption on the MIP-coated QCM sensor.

### 2.6. Gold-Coated QCM (Au) Sensor Regeneration

After the experiments, the gold-coated QCM (Au) sensor was regenerated in a piranha solution containing 60:40 (*v*/*v*) ammonium hydroxide (25%) and hydrogen peroxide (≥30%). The gold-coated QCM (Au) sensor was regenerated for 30 min. After regeneration, the piranha solution was cleaned with distilled water 3 times. The sensor was left to dry naturally before the next use. After regeneration, sensors are stored in a 50:50 (*v*/*v*) distilled water–acetonitrile mixture.

All experiments were repeated at least 2 times.

## 3. Results

To develop an effective and stable molecularly imprinted polymer (MIP), it is important to understand the analyte’s interactions with the polymer surface. In normal-phase mode, analytes interact with the MIP sorbent through shape- and size-selective interactions, enabling selective retention of target molecules while structurally dissimilar compounds are efficiently excluded. Elution is typically achieved by increasing the polarity or strength of the mobile phase to disrupt these interactions. In reverse-phase mode, commonly applied for aqueous samples, binding occurs predominantly through hydrophobic interactions, and a suitable solvent system can remove interfering compounds without compromising specific analyte–MIP binding [29].

In the initial phase of this study, polymer layers were synthesized using geraniol as the template molecule, demonstrating the feasibility of pyrrole-based MIPs for selective recognition of essential oil components [25,30]. These imprinted layers provide a foundation for further investigation of the effects of layer architecture on adsorption behavior, interfacial stability, and regeneration characteristics.

The study further compared single-layer and double-layer MIP configurations. It should be noted that the total number of electropolymerization cycles differs between the two architectures, leading to thicker polymer films in the double-layer configuration. Consequently, direct comparison of absolute EQCM frequency shifts in terms of adsorption capacity may be influenced by film thickness and polymer volume. Therefore, enhanced frequency responses are not interpreted solely as evidence of increased binding capacity. Instead, emphasis is placed on comparative trends in signal stability, adsorption reversibility, and response evolution during repeated adsorption–desorption cycles, which are less sensitive to thickness effects and more indicative of the stabilizing role of the underlying non-imprinted layer.

This study focuses on the mechanistic evaluation of single- or double-layer MIPs, specifically electropolymerization, interfacial behavior, and stability of the polymer layer. Quantitative analytical performance metrics such as linear range, sensitivity, calibration slope, detection and quantification limits, repeatability, reproducibility across larger sample sets, and service life were not assessed in this work. These parameters are beyond the scope of the present mechanistic study and will be addressed in future work aimed at developing fully validated analytical sensors.

### 3.1. Single-Layer Polymer Configuration

**Figure 1:**
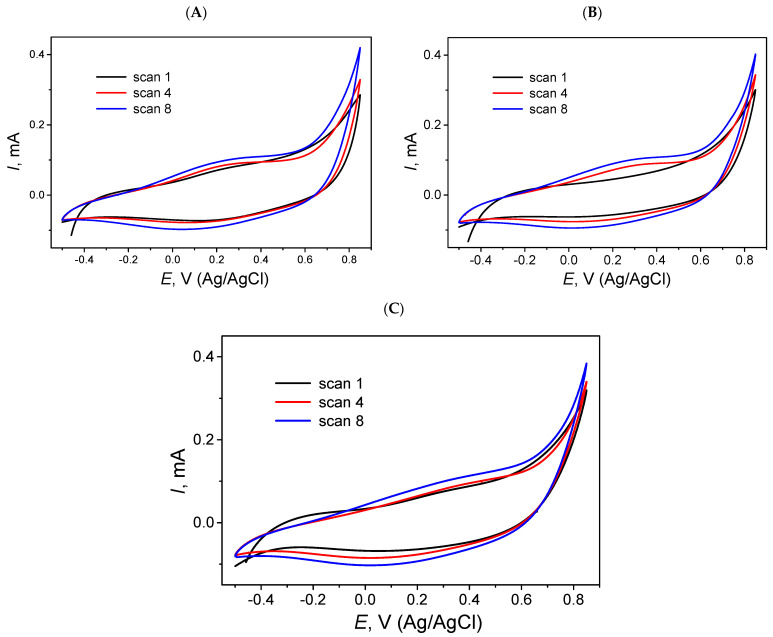
Electropolymerization processes on the QCM (Au) sensor surface. (**A**) Polymerization of the III solution on the QCM (Au) sensor surface, shown by CV over 8 consecutive cycles within a potential range of −500 mV to +850 mV at a scan rate of 50 mV s^−1^. (**B**) Electropolymerization of the double-layer MIP: formation of the base layer using the III solution, deposited via CV over a potential range of −500 mV to +850 mV at a scan rate of 50 mV s^−1^ for 7 cycles. (**C**) Electropolymerization of NIP: formation of the base layer using the II solution, deposited via CV over a potential range of −500 mV to +850 mV at a scan rate of 50 mV s^−1^ for 7 cycles.

Single-layer MIP films were generated by electropolymerization of pyrrole in the presence of geraniol using CV. The polymerization was carried out in Solution III containing pyrrole and geraniol as the template, using a potential range of −500 to +850 mV at a scan rate of 50 mV/s over 8 cycles (Figure 1A). The number of cycles was chosen to ensure continuous and mechanically stable film formation rather than to achieve a specific thickness, as the focus of this study is on interfacial behavior and layer properties.

**Figure 2:**
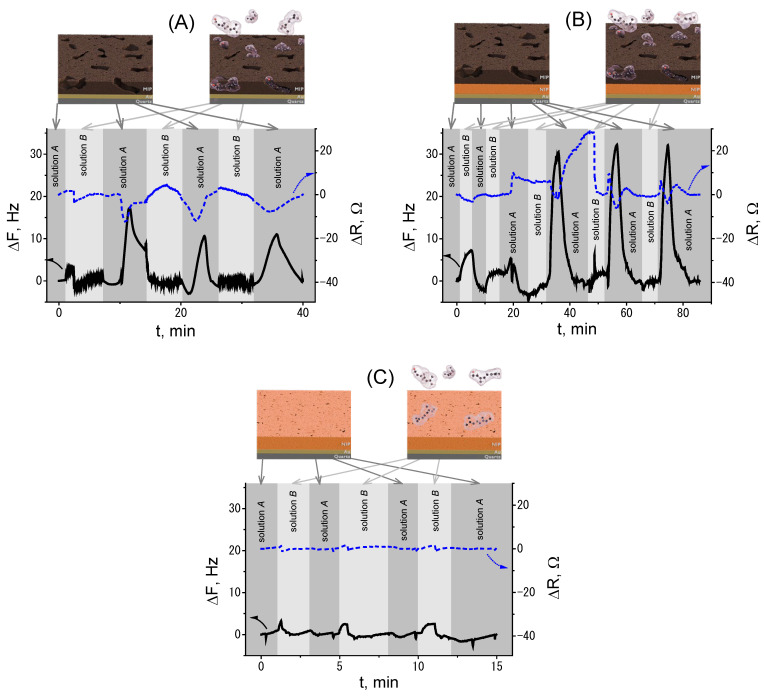
QCM response of different polymer layers toward Solution B. The solid black line represents the change in the ΔF value of the QCM sensor, while the dotted blue curve represents the change in the resistive element ΔR of the QCM sensor. (**A**) QCM measurement of the single-layer MIP at a flow rate of 1 mL min^−1^. The rectangle highlights the response to Solution B. (**B**) QCM measurement of the double-layer MIP at a flow rate of 1 mL min^−1^. The rectangle indicates the response to Solution B at 100% concentration exposure. (**C**) QCM measurement of the NIP on the QCM (Au) sensor at a flow rate of 1 mL min^−1^. The rectangle marks the response to Solution B at 100% concentration exposure.

Following electropolymerization, the electrode surface darkened slightly, indicating polymer formation. CV data (Figure 2A) showed reproducible oxidation peaks across successive cycles (+650 to +850 mV), with peak currents increasing from cycle 1 to cycle 8, confirming progressive growth and stabilization of a conductive polymer layer.

CV analysis (Figure 1A) demonstrated reproducible oxidation peaks across successive cycles, consistent with progressive film growth and stabilization. Comparison between cycle 1 and cycle 8 showed an increase in peak current, reflecting continuous modification of the electrode surface and successful formation of an electroactive polymer layer.

EQCM measurements were performed to investigate the interfacial response of the single-layer MIP to geraniol solutions at relatively high concentrations (0.025–0.05 mol·L^−1^), which were chosen to ensure measurable frequency shifts for mechanistic evaluation (Figure 2A). These measurements are intended to probe interfacial and electrochemical behavior rather than to determine analytical performance metrics such as sensitivity or detection limits.

The frequency response of the single-layer MIP showed gradual changes across repeated adsorption–desorption cycles, with some variability and partial signal recovery. This behavior is indicative of partial irreversibility, likely due to multilayer adsorption or temporary trapping within the polymer matrix. The sensor showed decreasing stability over time, leading to inconsistent ΔF and ΔR responses across repeated measurements. The relatively small frequency shifts (~1–2 Hz) are near typical instrument noise thresholds for many QCM setups, which helps explain why concentrations below 0.025 mol·L^−1^ were not reliably measured.

For illustration, a representative measurement at 0.05 mol·L^−1^ produced a frequency shift of approximately 1.36 Hz, corresponding to a measured mass uptake of 24.07 ng on the 0.20 cm^2^ active sensor area. The Sauerbrey Equation (1) was used [31]:

(1) $$\Delta m=\frac{-C\cdot \Delta F}{n}$$

where Sauerbrey mass sensitivity constant C = 17.7 ng·Hz^−1^ for a 5 MHz crystal and n = 1 (fundamental frequency). This enabled the estimation of mass loading and sensor response at a concentration at which QCM data were reproducible.

To quantify the surface coverage (Γ) [32] and the number of moles of geraniol bound to the sensor surface, the following Equation (2) was applied:

(2) $$\Gamma \;=\;\frac{\Delta m}{A}$$

where Γ is the surface coverage in grams per square centimeter, and A is the active sensor area (assumed to be 0.2 cm^2^).

Equation (3) was also applied:

(3) $$n\;=\;\frac{\Delta m}{MW}$$

where n is the amount of substance in moles, and MW is the molecular weight of geraniol (154.25 g/mol).

These values are summarized in Table 2. These calculations are provided solely to illustrate the magnitude of the interfacial response and are not intended as quantitative metrics of sensor performance.

Although adsorption modeling [33] (e.g., Freundlich or Langmuir–Freundlich) was initially considered [34,35], the variability observed across different concentrations indicates that extracting meaningful adsorption parameters was not feasible under the present conditions. Repeated measurements (n = 3) consistently showed similar trends, highlighting reproducible interfacial behavior and progressive stabilization of the single-layer polymer during successive cycling. For example, the relatively small resistance changes (ΔR) observed during measurements suggest limited viscoelastic losses (Figure 2A). Therefore, the Sauerbrey equation was used as a first-order approximation for mass evaluation. However, the calculated values should be considered as effective mass changes.

The results indicate that the single-layer MIP forms a conductive, electroactive polymer layer capable of reproducible interfacial responses to the template molecule. Variability and partial irreversibility in the EQCM data suggest that adsorption dynamics are influenced by local polymer density and film heterogeneity, which are common features of electropolymerized MIPs [22]. These findings provide mechanistic insight into how layer formation and surface characteristics govern interfacial stability and signal behavior, independent of absolute adsorption or analytical performance. Future work may include systematic variation of film thickness, polymerization parameters, and surface characterization (e.g., SEM or AFM) to further elucidate the relationship between polymer architecture and interfacial behavior.

### 3.2. Double-Layer Polymer Configuration

The polymerization process was conducted in two sequential stages. First, a base layer of pyrrole (NIP) was electrochemically deposited onto a chemically cleaned the QCM (Au) sensor surface to promote adhesion and uniform film formation. The resulting layer appeared as a uniform dark coating, with minor localized darker spots corresponding to regions of higher polymer density (Figure 1B).

In the second stage, a geraniol-imprinted MIP was formed on top of the base layer by electropolymerizing pyrrole in the presence of geraniol. This was performed using cyclic voltammetry over a potential range of −500 mV to +850 mV at a scan rate of 50 mV/s for 5 cycles. CV data showed a stepwise increase in oxidation peak current across successive cycles for both the base and top layers, indicating continuous polymer growth and successful incorporation of template-induced recognition sites (Figure 1B). The progressive increase in peak heights (+650 mV to +850 mV) confirms the controlled, layer-by-layer construction of the double-layer polymer.

EQCM measurements were performed to investigate interfacial behavior under mechanistic conditions using geraniol solutions at 0.025–0.05 mol·L^−1^ (Figure 2B). Representative measurements at 0.05 mol·L^−1^ yielded a frequency shift of −34 Hz and a resistance change of 3.5 Ω, indicative of a polymer–analyte interfacial response. Repeated adsorption–desorption cycles revealed partial irreversibility, likely due to multilayer adsorption or temporary trapping within the polymer cavities.

Repeated adsorption–desorption cycles revealed partial irreversibility of geraniol binding within the polymer matrix, likely due to multilayer adsorption or temporary trapping within polymer cavities. This behavior highlights the role of polymer architecture in governing interfacial interactions but also points to limitations in future sensor reusability and signal consistency. While the double-layer architecture increases the overall polymer volume, the improved signal stability and reproducibility suggest that the non-imprinted underlayer contributes to interfacial stabilization beyond mass effects.

The corresponding mass change, surface coverage, number of moles, and number of molecules adsorbed were calculated using the Sauerbrey equation and rounded to reflect experimental uncertainty (Table 3). The measured mass change was ~602 ng, corresponding to a surface coverage of ~3.0 × 10^−6^ g/cm^2^ (~3000 ng/cm^2^). The number of adsorbed moles was ~3.90 × 10^−9^ mol, equivalent to ~2.35 × 10^15^ molecules, or ~1.18 × 10^16^ molecules/cm^2^. These values are provided for illustrative purposes to quantify the magnitude of the interfacial response and are not intended as precise metrics of sensor performance.

Regeneration was evaluated by washing the sensors with Solution A. For the single-layer MIP (n = 3), the frequency response partially recovered after washing, but recovery was inconsistent and decreased with successive cycles (ΔF ranges: 0–18 Hz, −5 to 10 Hz, 0–11 Hz; Figure 2B). In contrast, the double-layer MIP (n = 3; Figure 2B) showed more consistent partial recovery, with ΔF values ranging from 5 to 28 Hz during washing cycles.

Recovery percentages were calculated relative to the initial adsorption frequency shift (ΔF_adsorption_). For the single-layer MIP, recovery ranged approximately 0–60% across successive cycles, showing high variability. The double-layer MIP exhibited higher and more consistent recovery (~50–80%), consistent with improved interfacial stabilization provided by the non-imprinted PPy underlayer. These results highlight that the double-layer architecture supports more reproducible polymer–analyte interactions during regeneration, although full desorption is not achieved under the applied washing conditions.

Nevertheless, full recovery was not achieved for either configuration, emphasizing that irreversible adsorption remains a limitation. This underscores the need to further optimize polymer design and regeneration protocols to enhance reusability and signal stability.

### 3.3. Non-Imprinted Polymer (NIP) Formation

To verify the functionality of the MIP sensor, a non-imprinted polymer (NIP) was synthesized under identical electrochemical conditions, but without the template molecule. Electropolymerization was performed using CV with a potential range of −500 mV to +850 mV, a scan rate of 50 mV/s, and 7 cycles (Figure 1C). The number of layers was matched to that of the MIP sub-layer to ensure consistent thickness and morphology. The resulting film appeared as a uniform brownish layer, serving as a reference for evaluating non-specific binding.

The NIP base layer, electropolymerized from 0.05 mol/L pyrrole, showed a progressive increase in oxidation peak current between +700 and +800 mV and a corresponding reduction response between −100 and +200 mV, confirming uniform polymer deposition during the seven-cycle formation of the NIP layer (Figure 2C).

QCM measurements with geraniol solutions showed minimal changes in resistance or frequency on the NIP sensor, confirming that the binding observed in the MIP is largely template-driven. However, no structurally similar interferents were tested, so the selectivity of the MIP sensor against potential interfering molecules remains to be validated in future studies.

### 3.4. Polypyrrole Film Thickness Evaluation

The polypyrrole film thickness was estimated from the QCM frequency shift using the Sauerbrey equation (Table 4). Frequency changes (ΔF) were converted to mass change (Δm) using the quartz sensitivity factor and sensor area. The resulting mass was then divided by the polypyrrole density (1.5 g/cm^3^) and the working surface (0.2 cm^2^) to obtain film thickness. Single-layer films were ~2.21 μm, whereas double-layer and NIP films were ~0.28–0.29 μm.

The larger frequency change observed for the single-layer film corresponded to a greater mass deposition, consistent with the Sauerbrey relation. The similar thicknesses of the double-layer and NIP films suggest that the imprinting process did not substantially increase the overall film mass, although it may influence the structural or morphological properties of the film. These results highlight the impact of deposition method on film thickness, which may, in turn, affect sensor sensitivity and response behavior. The measurements were performed on a consistent working surface area (0.2 cm^2^), allowing direct comparison between different film types and providing insight into their deposition efficiency.

## 4. Discussion

In our study, geraniol was observed to adsorb onto the MIP-coated sensor surface, consistent with the formation of specific recognition sites.

The adsorption of geraniol (Ger) onto MIP, NIP, or double-layer MIP surfaces—that is, onto different layers of polypyrrole (PPy)—can be described by the following general reaction equation:

PPy_suf_ + Ger ⇆ PPy_suf_ Ger (4)

In other words, it can be said that during this reaction, a surface complex compound of polypyrrole and geraniol, PPy_suf_ Ger, is formed. In this reaction, PPy_suf_ corresponds to the so-called active sites of the polypyrrole surface layer, i.e., the most energetically favorable sites on the surface to which geraniol molecules can adsorb. In the case of MIP and double-layer MIP, PPy_suf_ is expected to correspond to the cavities in the MIP structure itself. In this case, (4) the reaction rate should depend on both the surface concentrations or amounts (moles) of geraniol and PPy_suf_. Therefore, the reaction rate could be described by second-order kinetics. Since the QCM method measures a change in mass that is proportional to the amount of material, we assumed that the amounts of geraniol and PPysuf in our experiment were equal, i.e., *n*(Ger) = *n*(PPy_suf_). Therefore, applying the laws of second-order reaction kinetics, the amount of the reaction product PPy_suf_ Ger during the adsorption of geraniol could change over time *t* according to the following equation [36]:

(5) $$n({PPy}_{suf}Ger)=\frac{{k_{ads\;}\times \;n(Ger)}_{o}\;\times \;t\;}{1+k_{ads\;}\times \;t}$$

where, in this case, *k_ads_* is the rate constant of the second-order direct reaction (4), and *n*(Ger)_o_ is the initial amount of geraniol (and PPy_suf_).

On the other hand, during the desorption of geraniol, i.e., the reversible (4) reaction, its rate depends solely on the surface concentration of PPy_suf_ Ger, *n*(PPy_suf_ Ger). This reaction should follow first-order kinetics. Therefore, during the desorption of geraniol, *n*(PPy_suf_ Ger) could change over time *t* according to the following equation [36]:

(6) $$n({PPy}_{suf}Ger)={n({PPy}_{suf}Ger)}_{o}\times e^{-k_{des}\;\times \;t}$$

where, in this case, *k*_des_ is the rate constant of the first-order reverse reaction (4), and *n*(PPy_suf_ Ger)_o_ is the initial concentration of surface complexes.

To determine the rates of geraniol adsorption and desorption, i.e., *k_ads_* and *k*_des_, we applied Equations (5) and (6). Since the signal recorded by QCM, the change in resonance frequency Δ*F*, is proportional to the change in electrode mass Δ*m*, which in turn is proportional to the amount of geraniol adsorbed and, consequently, to the amount of PPy_suf_ Ger formed *n*(PPy_suf_Ger), Equations (5) and (6) can be modified by multiplying them by a certain proportionality constant *φ* (mol/Hz), as demonstrated in our previous study [25].

**Figure 3:**
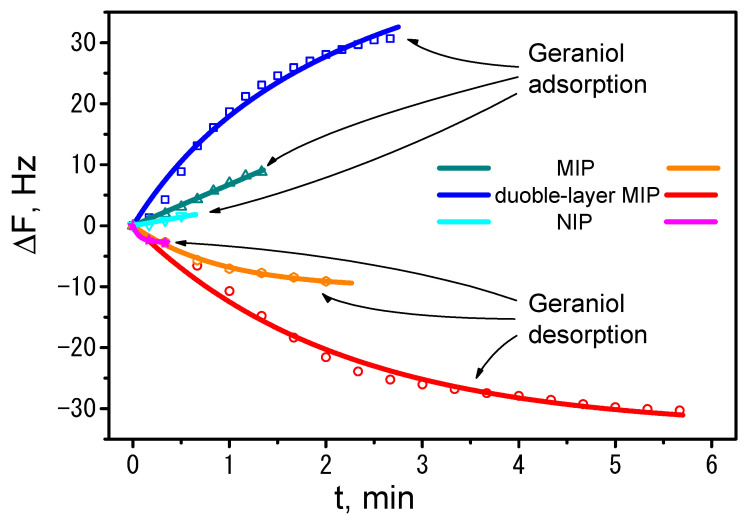
The adsorption and desorption kinetics data for geraniol, calculated from the Δ*F* curves shown in Figure 2 are plotted as data points (figures). The theoretical Δ*F* curves calculated using Equations (5) and (6) are shown as solid lines.

In the next stage of data processing, we applied the Δ*F* changes shown in Figure 2 when testing the sensitivities of the MIP, double-layer MIP, and NIP layers to geraniol adsorption. The figure shows the adsorption (green, blue, and light blue) and desorption (orange, red, and purple) rates of geraniol. These data were calculated by averaging three experiments (shown as points). We then performed a fit on this data, applying Equation (5) to the adsorption data and Equation (6) to the desorption data. Solid lines in the corresponding colors represent the data shown above (Figure 3).

After performing the rate-fitting procedure for the adsorption–desorption of geraniol, the rate constants *k*_ads_ and *k*_des_ were determined and are shown in Table 5. From this data, we were able to estimate the association constants *K*_asoc_ of geraniol with different PPy surfaces, which were calculated using the following equation:

(7) $$K_{asoc}=\frac{k_{ads}}{k_{des}}$$

From the *K*_asoc_ values presented in Table 5, we can see that the highest interaction of geraniol with the PPy surface was observed in the case of the double-layer MIP. Meanwhile, as noted earlier, the interaction with the NIP surface is minimal. When comparing the interactions of geraniol with a single-layer MIP and a double-layer MIP, a clear difference is also evident. This once again confirms our initial conclusions that geraniol partially blocks the Au surface, thereby preventing the formation of a PPy layer. Therefore, to obtain a more effective geraniol MIP layer, we should first deposit a pure PPy layer on the Au surface, thereby facilitating the subsequent MIP layer synthesis.

QCM data indicate that adsorption is partially irreversible, likely due to multilayer trapping or strong affinity within polymer cavities. This behavior underscores the influence of polymer architecture on interfacial interactions, but also highlights limitations in sensor reusability and reproducibility.

**Figure 4:**
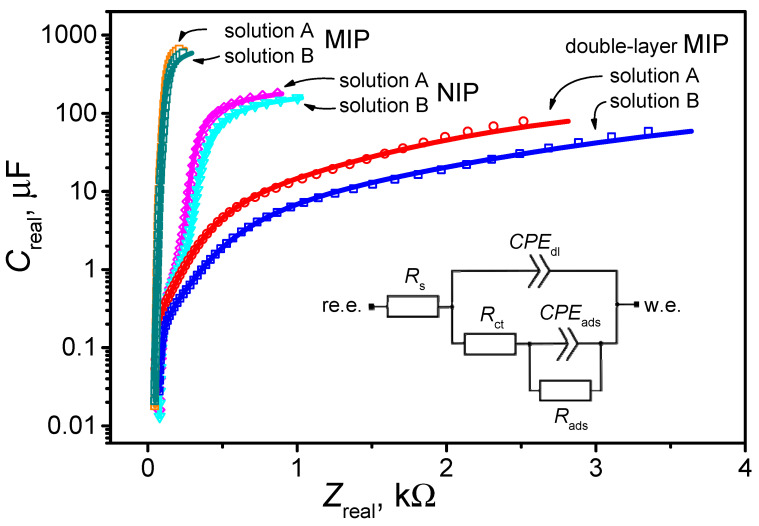
The EIS was recorded using the working Au electrode of a QCM sensor coated with different PPy layers: MIP, double-layer MIP, and NIP. The data were recorded in solution A and solution B. The solid lines represent the fitted curves obtained using the equivalent circuit shown.

Electrochemical impedance spectra (EIS) were recorded in a three-electrode QCM cell. Figure 4 shows the main data, represented by points (figures). To analyze the data, we used a modified Randles equivalent circuit, as shown in Figure 4 [37]. In this circuit, *R*_s_ represents the solution resistance, *CPE*_dl_ represents the double-layer capacitance (constant-phase element), *R*_ct_ represents the charge-transfer resistance, *CPE*_ads_ represents the adsorption capacitance (constant-phase element), and *R*_ads_ represents the adsorption resistance.

Table 6 presents the equivalent parameters obtained from a digital fitting procedure applied to all EIS data. The table does not include *R*_s_ data because an electrolyte of similar composition was used in all experiments; consequently, the measured solution resistance ranged between 42 and 71 Ω. It was also found that *R*_ads_ was greater than 1 GΩ in all cases, so this value was negligible.

From the EIS spectra shown in Figure 4 and the data in Table 6, it can be observed that in all cases—i.e., MIP, double-layer MIP, and NIP—the capacitance of the double electric layer decreases in the presence of geraniol in the solution (Solution B). This effect was most significant on the double-layer MIP polypyrrole layer, where the *CPE*_dl_ was reduced by a factor of 2.5. Meanwhile, for a simple MIP and NIP, the ratio is approximately 1.4. This confirms the QCM data and shows that geraniol is most likely to interact with the double-layer MIP layer.

Regeneration experiments demonstrated that frequency recovery was incomplete for both single- and double-layer MIPs, with partial stabilization afforded by the non-imprinted base layer in the double-layer configuration. These findings indicate that further optimization of polymer architecture and washing protocols is required to improve signal recovery.

The present work was conducted at relatively high geraniol concentrations (0.025–0.05 mol·L^−1^) and did not include testing against structurally similar interferents. Therefore, conclusions regarding sensor sensitivity and practical application in EOs analysis remain preliminary. Detailed site-specific adsorption and LOD studies will be addressed in future work.

Conceptually, the observed retention of geraniol suggests that MIP layers could be explored for applications beyond simple sensing, such as controlled capture and potential release of target molecules. Realizing such applications would require further study of polymer design, environmental conditions, and signal transduction methods.

Overall, these findings provide mechanistic insight into how polymer architecture governs adsorption, stabilization, and partial regeneration, establishing a foundation for future development of MIP-based electrochemical sensors with improved performance.

## 5. Conclusions

Pyrrole-based MIP films were fabricated to bind geraniol, as confirmed by CV and QCM. The double-layer design improved partial recovery during regeneration, indicating enhanced interfacial stabilization compared to the single-layer MIP. Partial irreversible adsorption and high analyte concentrations (0.025–0.05 mol·L^−1^) highlight limitations in reusability and practical applicability. These results provide proof-of-concept for MIP-based recognition of geraniol and establish a foundation for future optimization.

## Figures and Tables

**Table 1:** Steps and parameters of MIP/NIP formation.

Step No.	Single-Layer MIP	Double-Layer MIP	NIP
**1**	CV using solution I performed over a potential window of −500 to +1000 mV, repeated for four cycles at a sweep rate of 100 mV/s.		
**2**	Polymerization using solution III was conducted between −500 mV to +850 mV, with a scan rate of 50 mV/s, and a total of 8 cycles, to ensure polymer layer growth while capturing film formation dynamics.	Polymerization was implemented in two steps: Solution II: −500 and +850 mV, scan rate of 50 mV/s, and 7 cycles. Solution III: −500 mV to +850 mV, a scan rate of 50 mV/s, and 5 cycles.	Polymerization using solution II was conducted between −500 and +850 mV, scan rate of 50 mV/s, with 7 cycles at the same sweep rate, to ensure polymer layer growth while capturing film formation dynamics.

**Table 2:** Quantitative analysis of geraniol adsorption on the MIP-modified QCM electrode at 0.05 mol·L^−1^.

Parameter	Symbol/Formula	Value
Measured mass change	Δm	24 ng (2.4070 × 10^−8^ g)
Frequency shift (magnitude)	|ΔF| = Δm/C	1.36 Hz
Surface coverage	Γ = Δm/A	1.20 × 10^−7^ g·cm^−2^ (120.35 ng·cm^−2^)
Moles adsorbed	n = Δm/MW	1.56 × 10^−10^ mol
Total molecules adsorbed	N = n × N_A_	9.40 × 10^13^ molecules
Molecules per unit area	N/A	4.70 × 10^14^ molecules·cm^−2^

Constants: Sauerbrey mass sensitivity constant, C = 17.7 ng·Hz^−1^; active sensor area, A = 0.20 cm^2^; molecular weight, MW = 154.25 g·mol^−1^; Avogadro constant, N_A_ = 6.02214076 × 10^23^ mol^−1^.

**Table 3:** Quantitative Analysis of Geraniol Adsorption on Double-Layer MIP.

Parameter	Symbol/Formula	Value
Measured mass change	Δm	~602 ng (6.02 × 10^−7^ g)
Frequency shift (magnitude)	ΔF	−34 Hz
Surface coverage	Γ = Δm/A	~3.0 × 10^−6^ g/cm^2^ (~3000 ng/cm^2^)
Moles adsorbed	n = Δm/MW	~3.90 × 10^−9^ mol
Total molecules adsorbed	N = n × N_A_	~2.35 × 10^15^ molecules
Molecules per unit area	N/A	~1.18 × 10^16^ molecules/cm^2^

Constants: Sauerbrey mass sensitivity constant; active sensor area, A = 0.20 cm^2^; molecular weight, MW = 154.25 g·mol^−1^; Avogadro constant, N_A_ = 6.02214076 × 10^23^ mol^−1^.

**Table 4:** Frequency Response and Calculated Thickness of Polypyrrole Coatings.

Experiment Type	ΔF (Hz)	Δm (g)	Thickness h (μm)
Single-layer	18.75	6.63 × 10^−5^	2.20
Double-layer	2.34	8.27 × 10^−6^	0.28
NIP	2.45	8.67 × 10^−6^	0.29

**Table 5:** The rate constants *k*_ads_ and *k*_des_.

	*k_ads_*	*k_des_*	*K*_asoc_
MIP	7.58 × 10^−4^ ± 0.019	1.09 ± 0.028	6.95 × 10^−4^
double-layer MIP	0.420 ± 0.0215	0.469 ± 0.008	0.895
NIP	3.35 × 10^−4^ ± 0.216	14.5 ± 0.835	2.31 × 10^−5^

**Table 6:** Equivalent parameters obtained from a digital fitting procedure applied to EIS data.

	Solution	*CPE*_dl_, μS·s*^α^*	*α*_dl_	*CPE*_ads_, μS·s*^α^*	*α*_ads_	*R*_ct_, Ω
MIP	A	389 ± 203	0.625 ± 0.067	559 ± 147	0.862 ± 0.045	59.7 ± 12.7
	B	272 ± 155	0.598 ± 0.069	596 ± 119	0.812 ± 0.035	54.1 ± 9.55
double-layer MIP	A	2.30 ± 1.39	0.841 ± 0.057	188 ± 11.5	0.445 ± 0.015	144 ± 37.7
	B	0.91 ± 0.78	0.915 ± 0.079	153 ± 9.32	0.424 ± 0.016	129 ± 57.7
NIP	A	34.7 ± 7.96	0.639 ± 0.027	225 ± 13.0	0.792 ± 0.017	220 ± 12.1
	B	23.9 ± 4.64	0.677 ± 0.023	207 ± 10.7	0.783 ± 0.017	261 ± 12.4

## Data Availability

The original contributions presented in this study are included in the article. Further inquiries can be directed to the corresponding author U.P.

## Abbreviations

CV	Cyclic voltammetry
EOs	Essential oils
EQCM	Electrochemical quartz crystal microbalance
MIPs	Molecularly imprinted polymers
NIP	Non-imprinted polymer
PPy	Polypyrrole
QCM	Quartz crystal microbalance
